# Reliability and validity of miniscrews as references in cone-beam computed tomography and intraoral scanner digital models: study on goat heads

**DOI:** 10.1186/s12903-019-0952-9

**Published:** 2019-11-27

**Authors:** Yiran Jiang, Gui Chen

**Affiliations:** Department of Orthodontics, Peking University School and Hospital of Stomatology, National Engineering Laboratory for Digital and Material Technology of Stomatology, Beijing Key Laboratory of Digital Stomatology, 22 Zhongguancun South Street, Beijing, 100081 China

**Keywords:** Orthodontic miniscrews, Digital dental models, CBCT, Reliability, Validity

## Abstract

**Background:**

Miniscrews have been used to superimpose three-dimensional (3D) craniofacial images as well as explore stable structures in jaws. Our purpose was to evaluate the reliability and validity of linear and angular measurements made with miniscrews on a 3D cone-beam computed tomography (CBCT) at two voxel sizes and compared to models created by an intraoral scanner (IOS).

**Methods:**

Altogether, 64 miniscrews were placed in 12 goat jaws. The jaws were scanned by CBCT machine at 0.12 mm and 0.3 mm voxels and by the IOS. Linear and angular measurements between miniscrews on CBCT at the two voxel settings and the IOS were compared with actual measurements and with each other.

**Results:**

An intra-and inter-class correlation of 0.961–1.000 were obtained by each method. Linear measurements showed significant overestimations of 0.27 ± 0.24, 0.14 ± 0.22 and 0.15 ± 0.26 mm, and angular measurements showed non-significant differences of 0.11 ± 1.97°, 0.15 ± 2.79° and 0.41 ± 2.34° for the CBCT at 0.12-mm, 0.3-mm voxels and the IOS, respectively. Equal magnification of linear measurements was on homolateral and contralateral sides using CBCT, whereas significantly greater magnification on the homolateral side than on the opposite was observed using the IOS. There was no significant difference with angular measurements between digital CBCT models at two voxels and IOS. In addition, all angular measurements were comparable to actual measurement results.

**Conclusions:**

Miniscrews in CBCT and IOS are reliable and clinical valid when used as a reference measuring tooth movement. However, when miniscrews are involved in high precision measurement in CBCT or IOS image, systematic error should be taken into consideration. When comparing CBCT images, using the same voxel size is recommended for miniscrew related measurements to reduce error.

## Background

Traditional orthodontic records including plaster dental models, facial and intra-oral photos, panoramic radiographs and lateral cephalograms could be used to monitor treatment progress and outcomes. Superimposing serial cephalograms has been used widely to determine the skeletal and dental changes that occur over time. Stable structures are the keys to a good superimposition. These structures described in Melsen’s research of cranial base growth [1], Bjork and Skieler’s implant research [2, 3], as well as Enlow’s investigation of remodeling [4] are also suggested by American Board of Orthodontics. The locations of these natural stable structures in maxilla and mandible were best found with external metal implant references, and the superimposition of serial cephalograms on metallic implants is considered to be the best technique.

Increasing developments in acquisition of medical images and 3D digital technologies have initiated revolutionary changes in orthodontics. Of recent, CBCT, digital dental models and 3D facial photos have become popular orthodontic records. The reliability and validity of these digital records have to be verified before they are used to make diagnosis and treatment plan. Similar to 2D cephalometric superimposition, orthodontists have tried to register serial 3D digital models to monitor treatment changes over time in three-dimensions. And a great number of studies have focused on CBCTs and digital dental models superimposition.

CBCT has been proven to be a valid 3D representation of the skull that is suitable for clinical and laboratorial usage. It is not difficult to superimpose non-growing patients’ serial CBCT models because several stable craniofacial structures can be used as references [5–7]. However, it is still challenging to do so on growing patients because 3D stable structures in jaws have not been identified. Superimposing on external references will be necessary to analyze changes in jaws of growing patients [8]. Parton et al. [9] attempted to superimpose mandibular structures in growing rabbits with the aid of implants. Nguyen et al. [10] identified stable mandibular structures in three dimensions in growing patients with the aid of bone plates.

Recent decades have also witnessed remarkable advancements in digital dental model technologies, from stone dental model scanning to direct intraoral scanning. Digital software makes superimposition of serial dental models possible. Palatal rugae have historically been used to perform 2D measurements on 3D dental models [11–13]. With the aid of miniscrews, Jang et al. [14] and Chen et al. [15] evaluated the stability of the palatal region and established a 3D superimposition method for analyzing orthodontic tooth movement in maxillary dental models, respectively. However, it is still unknown how to superimpose serial digital dental models in growing patients, and again, metallic implants such as miniscrews could be identified as external references in a future study. Beforehand, the positional stability of miniscrews during orthodontic treatment should be evaluated, because only stable miniscrews could be used as references. The linear distance and angle measurements between miniscrews are two methods applied in previous studies [8, 16].

What calls for noteworthy attention is that studies showed that artifact caused by the metallic implant in CBCT will degrade image quality [17], which could bring errors into the procedure of implant superimposition. Park et al. [18] also found that the borders of metal brackets were blurred in image created by certain type of IOS. Another literature disagreed with the use of IOSs for impression capture of multiple dental implants, aimed at the manufacture of extended implant-supported restorations as full arches [19]. Previous studies have confirmed the reliability and accuracy of digital images about anatomy on jaws bones or dentition by comparing the linear distance between landmarks on digital images with actual values [20–27]. However, no study has quantified the systematic error of digital miniscrew images.

The aim of this study is to evaluate the reliability and validity of linear and angular measurements of miniscrews in CBCT at different voxel sizes and IOS. This was the first attempt to quantify the systematic errors of miniscrew images and test the reliability of miniscrew measurements on CBCT and IOS, and we hope that the result could be served as justification for further evaluation of miniscrew stability and application of miniscrew superimposition on 3D models.

## Methods

**Fig. 1:**
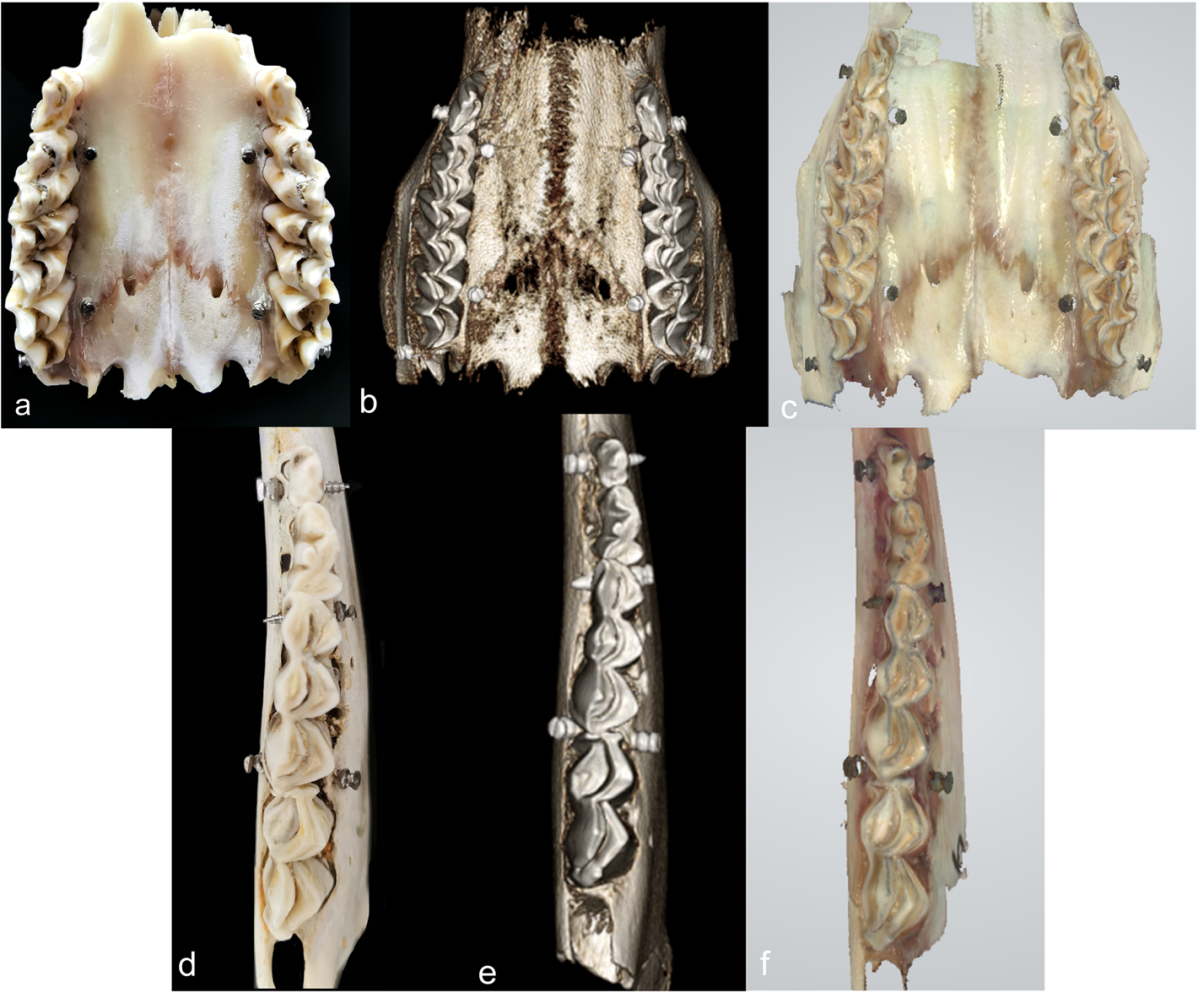
**a** and **d** Two representative images among 12 samples. **b** and **e** 3D models originating from CBCT of the two actual samples on the left side. **c** and **f** 3D models of the same samples on the left scanned from the IOS

Four goat maxillae and four mandibles were obtained from the agricultural market for human daily consumption. The goats had already been sacrificed at the time of purchase. For this experiment and under these conditions, the research did not require approval from the regional ethical committee for research ethics due to national legislation. The lower jaw was dissected further into two hemi-mandibles to make direct scanning possible. Maxillae and hemi-mandibles underwent miniscrew (11 mm × 1.6 mm; Ci Bei, Zhejiang, China) implantation by two experienced orthodontists. Two miniscrews were placed on the buccal and lingual sides of each maxilla and hemi-mandible. At least one miniscrew on each hemi-mandible penetrated out of the cortical bone from one side to another (Fig. 1a, d). In all, 64 miniscrews were inserted.

### CBCT and intraoral imaging

**Fig. 2:**
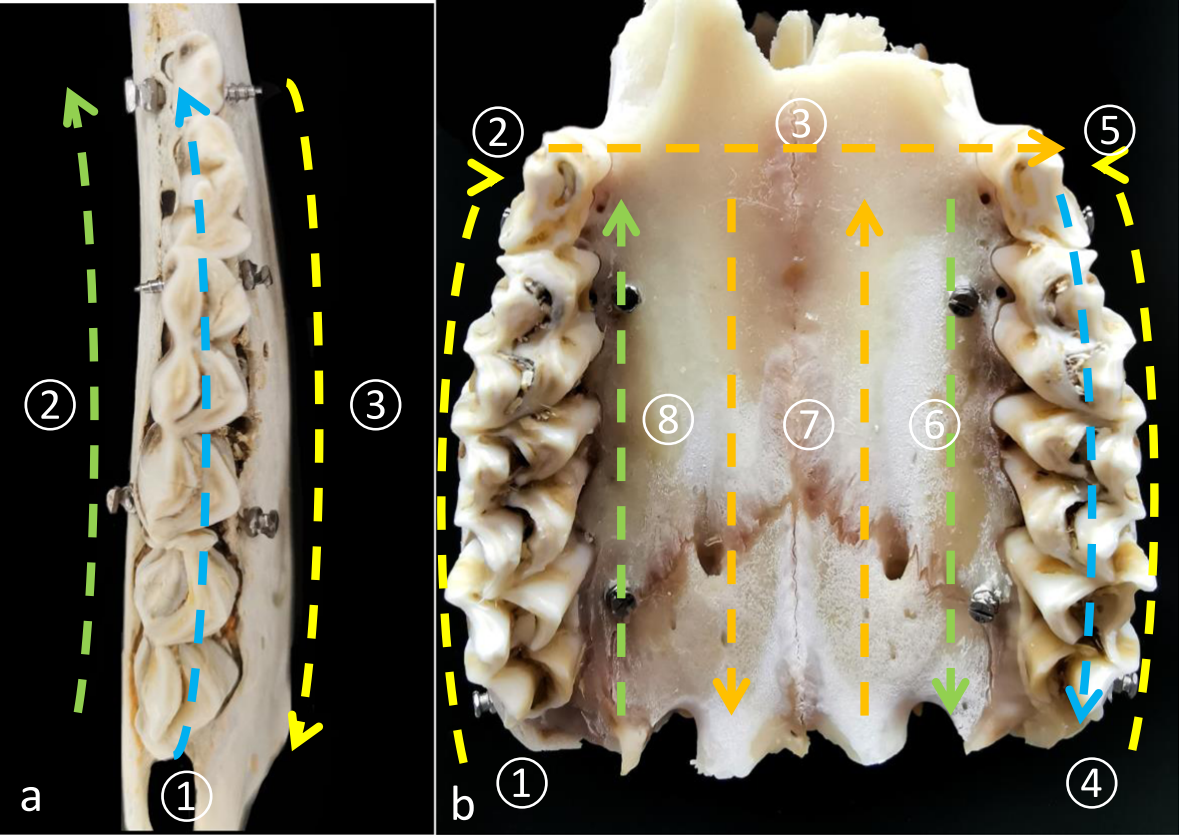
The imaging sequence of the IOS. **a** Representative imaging sequence for hemimandible samples: occlusal–buccal–lingual. **b** Representative imaging sequence for maxillary samples: right occlusal–right buccal–anterior palatal–left occlusal–left buccal–left palatal–palatal–right palatal

All the samples were scanned by a NewTom GIANO system (Aperio, Sarasota, FL, USA) with a field of view of 11 cm × 11 cm × 5 cm and high resolution of 0.12-mm voxels. Eight hemi-mandibles were rescanned by 0.3-mm voxels. Invivo™ 6.0 (Anatomage, San Jose, California, USA) was used to generate 3D models by the preset threshold value of bone (Fig. 1b, e). A 3Shape TRIOS IOS (3Shape Dental Systems, Copenhagen, Denmark) using a regular calibration procedure was applied for imaging in vitro. The imaging sequence is depicted in Fig. 2. The 3D models were imported into RapidForm™ 2006 (INUS Technology, Seoul, Korea) for measurement.

### Linear and angular measurement of miniscrews

**Fig. 3:**
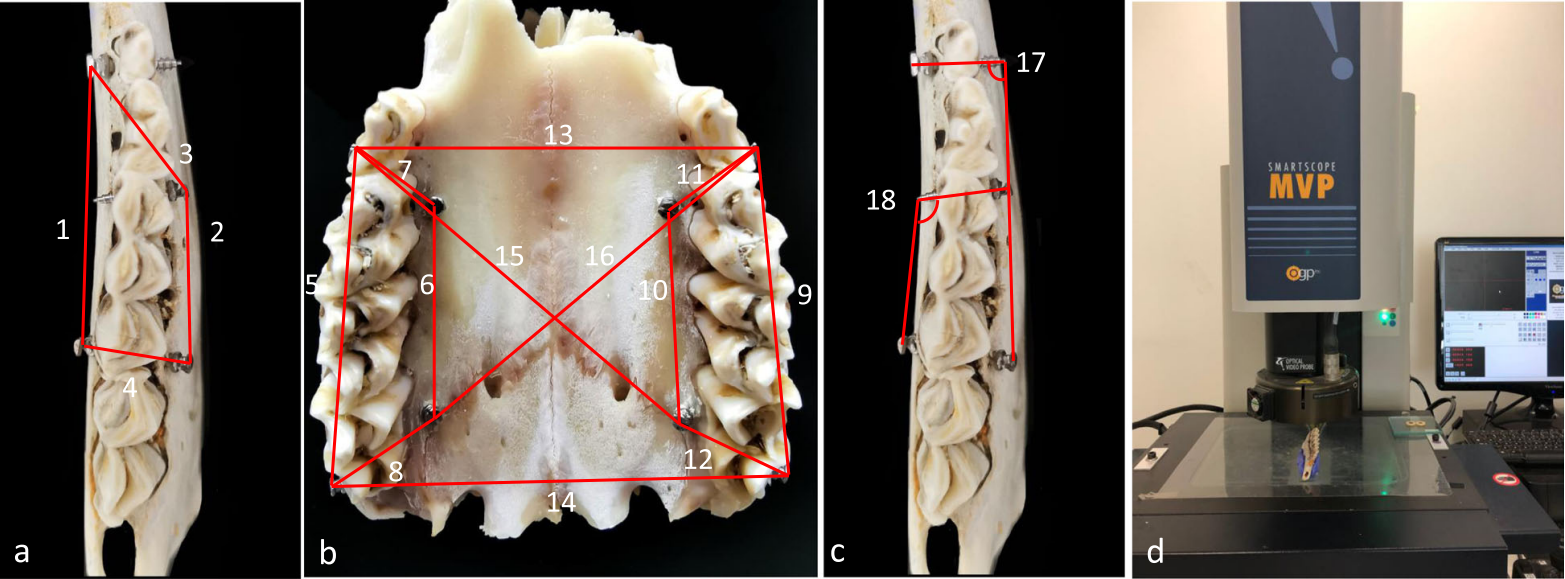
**a** and **b** Sixteen linear distances measured between two miniscrew heads on hemimandibles and maxillae. **c** angles measured on the hemimandible. **d** Smartscope MVP for actual measurement of angles

Measurements were undertaken by a single operator thrice for each sample on three digital models and a digital caliper (Airaj, Tsingtao, China) on real miniscrews. They were re-measured once by another operator to test inter-operator reliability. The surface center of the head and apex of each miniscrew were used as reference points. The point-to-point distance along a line was used as a linear measurement value (Fig. 3a, b). A measurement was abandoned if either of the points could not be set stably using a caliper pointer. A visual measurement system, SmartScope® MVP (OGP, Singapore), was used to measure the angle between real miniscrews (Fig. 3d). The angle had to consist of two ultimate points of one cortically penetrated miniscrew, and the third point was a surface center point of another miniscrew head or the apex of the miniscrew depending on which one was visually clear (Fig. 3c). Half of the cap of the miniscrews was ground off using a high-speed handpiece to allow better identification of reference points.

### Statistical analyses

Measurements taken by the digital caliper and Smartscope MVP on real miniscrews were considered to be real values. SPSS v25 (IBM, Armonk, NY, USA) was employed for statistical analyses. Intra- and inter-operator reliabilities were tested by intra-class correlation analysis (ICC). Then, the arithmetic mean value was calculated and used as the value of each measurement. All mean data were tested to follow the normal distribution. Therefore, paired t-test was conducted to evaluate the validity of measurements of miniscrews in CBCT and intraoral imaging. The level of significance was set at *P* < 0.01 for ICC and *P* < 0.05 for paired t-tests. An acceptable error for linear measurement for clinical application was set as ≤ ±0.5 mm [28]. Also, ≤ ±5° was deemed to be clinically acceptable for measuring systematic differences for angles [16].

## Results

Seventy-five paired linear measurements (35 homolateral measurements and 40 contralateral measurements) at 0.12-mm voxels, 31 linear measurement (20 homolateral measurements and 11 contralateral measurements) at 0.3-mm voxels and 11 angles were evaluated. Intra- and inter-operator reliability using ICC was 0.961–1.000.

Linear measurements by 3D models from CBCT at 0.12-mm voxels (termed “CBCT1” in all Tables) and 0.3-mm voxels (CBCT2) demonstrated overestimates on homolateral and contralateral sides compared with the paired results using the digital caliper (Table 1). The mean biases were 0.31 ± 0.20 mm and 0.25 ± 0.28 mm at 0.12-mm voxels, and 0.11 ± 0.21 mm and 0.19 ± 0.26 mm at 0.3-mm voxels. All 95% confidence intervals (CIs) were > 0 mm but < 0.5 mm. The only significant difference using the IOS relative to digital caliper pairs was observed on homo-lateral linear measurements. An equal amplification effect on sagittal and transverse directions was revealed by a one-sample *t*-test at 0.12-mm voxels and 0.3-mm voxels of CBCT with caliper measurements, whereas a significant increased enlargement was observed on the homo-lateral side using the IOS (Tables 1, 2). In total, significantly larger results were observed compared with value obtained using digital caliper pairs. Also, results at 0.12-mm voxels using CBCT were significantly larger than all other values (Table 4).

**Table 1:** Paired *t*-test for comparing linear measurement values (mm) between the three digital models with values from the digital caliper

Measurement	Mean bias	Standard deviation	95% confidence interval	t	P
Homolateral side					
CBCT1	0.31	0.20	0.24 to 0.38	9.111	< 0.001
CBCT2	0.11	0.21	0.02 to 0.21	2.450	0.024
IOS	0.25	0.20	0.18 to 0.32	7.206	< 0.001
Contralateral side					
CBCT1	0.25	0.28	0.16 to 0.34	5.637	< 0.001
CBCT2	0.19	0.26	0.02 to 0.37	2.473	0.033
IOS	0.04	0.27	−0.04 to 0.13	0.986	0.330
Total linear measurements					
CBCT1	0.27	0.24	0.22 to 0.33	9.739	< 0.001
CBCT2	0.14	0.22	0.06 to 0.22	3.505	0.001
IOS	0.15	0.26	0.09 to 0.21	5.106	< 0.001

**Table 2:** One sample *t*-test for comparing the differences in mean linear measurement (mm) between homolateral and contralateral sides of CBCT and IOS with measurements using the digital caliper (the test value was zero)

Measurement	Mean bias	Standard deviation	95% confidence interval	t	P
CBCT1	0.08	0.30	−0.01 to 0.19	1.714	0.093
CBCT2	−0.10	0.37	−0.35 to 0.15	−0.910	0.384
IOS	0.25	0.31	0.15 to 0.35	4.912	< 0.001

Angle measurements revealed good validity among the three digital methods compared with true values (Table 3). Values for standard deviation and ranges of 95%CIs were large. Significant differences among the three digital models were not observed (Table 4).

**Table 3:** Paired *t*-test for comparing angle measurement (°) values between the three digital models with actual measurements

Measurement	Mean bias	Standard deviation	95% confidence interval	t	P
CBCT1	0.11	1.97	−1.21 to 1.44	0.192	0.852
CBCT2	0.15	2.79	−0.88 to 1.19	0.330	0.748
IOS	0.41	2.34	− 1.17 to 1.98	0.574	0.579

**Table 4:** Paired *t*-test for values of linear (mm) and angle (°) measurements among the three digital models

Measurement	Mean bias	Standard deviation	95% confidence interval	t	P
Linear measurements					
CBCT1–CBCT2	0.20	0.26	0.10 to 0.29	4.231	< 0.001
CBCT1–IOS	0.12	0.24	0.07 to 0.18	4.434	< 0.001
0.3-mm voxels–IOS	−0.02	0.32	−0.14 to 0.10	−0.363	0.719
Angle measurements					
CBCT1–CBCT2	−0.04	0.97	−0.69 to 0.61	−0.134	0.896
CBCT1–IOS	−0.29	2.79	−2.17 to 1.58	−0.346	0.737
CBCT2–IOS	0.49	1.20	−0.37 to 1.35	1.288	0.230

## Discussion

Superimposing orthodontic records at different time points has been used widely to determine the craniofacial changes. The cornerstone of superimposition is using stable structures. Identification of stable structures in jaws without having external references in growing patients is extremely challenging. In history, metal implants have been used as reference in 2D cephalograms to explore natural stable structure [2, 3]. In 3D era, implants should continually play a crucial role in CBCT [8–10] and digital dental model superimposition [14, 15]. However, metallic implants would produce artifacts both in CBCT and IOS images, which would degrade the image quality and introduce errors. In this study, the experimental animal skulls, which are more feasible and less expensive than human skulls, were used to evaluate the reliability and validity of linear and angle measurements of 3D miniscrews on CBCT and IOS with actual values. The results of our study are applicable on human skulls as well, because the goat heads are merely platforms for miniscrew implantation. Moreover, the study is ethically impossible to be conducted on patients because of the amount of radiation exposure necessary when taking CBCT at different resolutions.

Our study showed that statistically significant overestimations of linear measurements were obtained on CBCT both at 0.12 (0.27 ± 0.24 mm) and 0.3 (0.14 ± 0.22 mm) voxels compared with actual measurements. Our results are, to some extent, consistent with several studies. Moshfeghi et al. [20] using gutta-percha, reported an enlargement by 0.10 ± 0.99 mm in axial section and 0.27 ± 1.07 mm coronal section at 0.3 voxels. However, the values for standard deviation were greater than our data. Tolentino et al. [21] used silica markers, but they did not observe statistical difference among voxels at 0.25, 0.3 or 0.4 mm. Variable materials used as references in different studies may attribute to the contradiction between studies. Schulze et al. [29] pointed that an extreme artifact could be produced by titanium implants. Instead of upgrading resolution, they suggested a more sophisticated reconstruction algorithm for meaningful reduction of artifacts. Moreover, when using linear measurement to evaluate the stability of miniscrews, the systematic error should be taken into consideration.

Secondly, miniscrews at two voxel settings presented reliable and accurate results on angle measurements when compared with actual values. Our result supported the use of angular measurements acquired through miniscrews in clinical applications, which is important on measuring the angle stability of miniscrews after orthodontic loading [16].

CBCT is limited for evaluation of short-term treatment effects due to excess radiation exposure to the patients. Thus, chairside IOS is promising for this purpose. DeLong et al. [30] found that a smooth textured surface (such as the titanium miniscrews used in our study) could worsen the digitizing performance due to spectral reflection. However, our study confirmed the clinical reliability and validity of IOS for linear and angular measurements of miniscrews, which were consistent with other studies. However, these measurements were different with respect to systematic errors and their tendencies [22–27]. Our results supported that the evaluation of tooth movement on serial digital dental models from IOSs during growth or after orthodontic intervention is operable. In addition, we also found it quite interesting that the mean bias on the homolateral side was significantly larger than that on the opposite, implying unequal magnification in sagittal and transverse directions. Anh et al. [31] claimed that regions imaged later would generate more errors during configuration than regions imaged earlier. Thus, the scanning sequence could be one of the reasons for the unequal amplification effect observed in our study, and a modification is required when miniscrews are involved.

Above all, in accordance with results of literatures and this study, the following suggestions are proposed when miniscrews are used to superimpose 3D image: 1.The positional stability of miniscrews should be evaluated in order to ensure the reliability and clinical validity of the linear and angular measurements on 3D models. 2. The same CBCT machine with the same scanning settings is required when doing superimposition. 3. Systematic errors of miniscrew measurements on CBCT image and digital dental models acquired from IOS should be consider when stable structures are explored.

A limitation of this study is the exclusion of motion artifacts because this is an experiment on dry goat jaw bone. In addition, the study was conducted for a single experimental condition by testing systematic errors on a specific type of miniscrew, a single CBCT machine and one IOS. Whether the results of this study are suitable for other miniscrews, other CBCT machines at different voxel sizes, or other IOSs is not known.

## Conclusions

1. The linear and angular measurements produced using minicrews as a reference to measure tooth movement seem reliable and clinically valid in images generated by CBCT and IOS. However, when miniscrews are involved in high precision measurements in CBCT or IOS image, such as exploration of a stable region, systematic error should be taken into consideration.
2. Maintaining the same voxel size in CBCT images is suggested when miniscrews are set as reference to measure the changes in craniofacial structures.

## Data Availability

The full datasets used and analyzed during the current study are available on reasonable request from the corresponding author at chengui723@163.com.

## Abbreviations

3D: Three-dimensional
CBCT: Cone-beam computed tomography
CI: Confidence intervals
ICC: Intra-class correlation analysis
IOS: Intraoral scanner
